# Lyme Disease: Diversity of *Borrelia* Species in California and Mexico Detected Using a Novel Immunoblot Assay

**DOI:** 10.3390/healthcare8020097

**Published:** 2020-04-14

**Authors:** Melissa C. Fesler, Jyotsna S. Shah, Marianne J. Middelveen, Iris Du Cruz, Joseph J. Burrascano, Raphael B. Stricker

**Affiliations:** 1 Union Square Medical Associates, 450 Sutter Street, Suite 1504, San Francisco, CA 94108, USA; melissacfesler@gmail.com; 2 IGeneX Reference Laboratory, Milpitas, CA 95035, USA; jyotsna@aol.com (J.S.S.); icruz@igenex.com (I.D.C.); burraj69@gmail.com (J.J.B.); 3 Atkins Veterinary Services, Calgary, AB, T3B 4C9, Canada; middel@telus.net

**Keywords:** Lyme disease, *Borrelia burgdorferi*, Relapsing Fever *Borrelia*, *Borrelia miyamotoi*, tickborne disease, immunoblot

## Abstract

Background: With more than 300,000 new cases reported each year in the United States of America (USA), Lyme disease is a major public health concern. *Borrelia burgdorferi* sensu stricto (Bbss) is considered the primary agent of Lyme disease in North America. However, multiple genetically diverse *Borrelia* species encompassing the *Borrelia burgdorferi* sensu lato (Bbsl) complex and the Relapsing Fever *Borrelia* (RFB) group are capable of causing tickborne disease. We report preliminary results of a serological survey of previously undetected species of Bbsl and RFB in California and Mexico using a novel immunoblot technique. Methods: Serum samples were tested for seroreactivity to specific species of Bbsl and RFB using an immunoblot method based on recombinant *Borrelia* membrane proteins, as previously described. A sample was recorded as seropositive if it showed immunoglobulin M (IgM) and/or IgG reactivity with at least two proteins from a specific *Borrelia* species. Results: The patient cohort consisted of 90 patients residing in California or Mexico who met the clinical case definition of chronic Lyme disease. Immunoblot testing revealed that 42 patients were seropositive for Bbsl (Group 1), while 56 patients were seropositive for RFB (Group 2). Eight patients were seropositive for both Bbsl and RFB species. Group 1 included patients who were seropositive for Bbss (14), *B. californiensis* (eight), *B. spielmanii* (10), *B. afzelii/B. garinii* (10), and mixed infections that included *B. mayonii* (three). Group 2 included patients who were seropositive for *B. hermsii* (nine), *B. miyamotoi* (seven), *B. turicatae* (nine), and *B. turcica* (two). In the remaining Group 1 and Group 2 patients, the exact *Borrelia* species could not be identified using the immunoblot technique. Conclusions: Lyme disease is associated with a diverse group of *Borrelia* species in California and Mexico. Current testing for Lyme disease focuses on detection of Bbss, possibly resulting in missed diagnoses and failure to administer appropriate antibiotic therapy in a timely manner. The genetic diversity of *Borrelia* spirochetes must be considered in future Lyme disease test development.

## 1. Introduction

*Borrelia* spirochetes are a significant cause of disease worldwide. *Borrelia* species fall within the family Spirochaetaceae and are characterized as Gram-negative, helical bacteria moving via periplasmic axial filaments [1,2,3]. Currently, there are at least 53 known *Borrelia* species categorized into three groups: approximately 22 species fall within the Lyme Disease group (*B. burgdorferi* sensu lato, Bbsl), and approximately 29 fall within the Relapsing Fever *Borrelia* (RFB) group. These two groups contain agents of Lyme disease (LD) and relapsing fever (RF), respectively [4,5,6]. The remaining species fall within a third genetically distinct group, and these species remain unclassified and primarily associated with reptiles [4,5,7].

The Bbsl group comprises genetically diverse bacteria that are distributed worldwide primarily throughout the Northern hemisphere and are vectored by ixodid (hard) ticks [6,8]. In the United States of America (USA), LD is currently the largest vector-borne illness and causes an array of symptoms including musculoskeletal, neuropsychiatric, and cardiac problems and, on rare occasions, even death [9,10]. At least 11 Bbsl genospecies were identified in North America including Bbss, *B. americana*, *B. andersoni*, *B. bissettii*, *B. californiensis*, *B. carolinensis*, *B. garinii*, *B. kurtenbachii*, *B. laneii*, *B. mayonii*, and *B. spielmanii* [6,11]. 

The RFB complex spirochetes are likewise genetically diverse but are primarily vectored by argasid (soft) ticks and the human body louse [12,13]. They are widely distributed throughout much of the world, and they are a significant cause of illness on five out of seven continents [12,13]. RFB are endemic to the Western USA, Southwest Canada, parts of Mexico, Central and South America, the Mediterranean, much of Asia, and throughout Africa [12]. While the ecology and epidemiology of RFB in Africa is well understood, distribution of RFB outside the African continent is less well known [13]. Multiple species of RFB are reported to cause disease in humans, and two species are associated with high fatality rates: *B. duttonii,* which is found in East Africa, and *B. recurrentis*, which is widely distributed through much of the world including Africa, South America, Europe, and Asia [12]. In North America, *B. hermsii* and *B. turicatae* are the species most commonly reported [12]. 

RFB infection should be considered a major public health concern. Individuals infected with RFB can develop flu-like symptoms such as recurrent fevers, arthralgias, myalgias, headaches, and nausea, as well as more severe symptoms, such as central nervous system involvement [14,15]. Patients infected with certain RFB species such as *B. miyamotoi* are said to exhibit more significant symptoms than patients with Bbsl infection [14]. Dissemination of various RFB spirochetes into the bloodstream is said to be between 100 and 1000 times faster than Bbsl species, resulting in extensive morbidity and mortality [10,16,17]. Like many spirochetal infections, prompt antibiotic treatment of RFB infections results in a better clinical outcome, although treatment may sometimes trigger a severe Jarisch–Herxheimer reaction [18].

The symptoms caused by vector-borne pathogens including Bbsl and RFB spp. are not specific, and patients infected by either Bbsl or RFB may also be infected with other vector-borne pathogens such as *Babesia*, *Ehrlichia*, *Anaplasma*, *Rickettsia*, or *Bartonella*, resulting in overlapping symptoms that cannot be differentiated in terms of the individual pathogen [19]. Diagnosis and reporting of LD cases is based on insensitive serological detection of Bbss infection, and it fails to take into account the genetic diversity of spirochetes capable of causing LD and LD-like illness [1,20,21,22]. Although research into the diagnosis and treatment of RFB infection is limited in scope, a recent pilot study suggested that seroreactivity against both Bbsl and RFB could be widespread in California, thus complicating the diagnosis of LD in that state [15]. 

To assess the impact of testing limitations and to determine levels of exposure to Bbsl and RFB species in California and Mexico, we employed a recently developed modified Western blot procedure, termed the line immunoblot, which uses recombinant antigens from common strains and species of the Bbsl and RFB groups for the serological diagnosis of LD and RF [1,2]. Testing was conducted over an eight-month period in 2019 on patients with suspected tickborne disease. Our findings confirm that the serotype makeup of spirochetal exposure in the USA and Mexico appears to be more complex than acknowledged previously.

## 2. Materials and Methods

### 2.1. Patients and Data Collection

Our patient cohort was recruited from a medical practice located in San Francisco, CA, USA, specializing in the diagnosis and treatment of tickborne diseases. The Western Institutional Review Board (WIRB), Puyallup, WA approved the anonymous retrospective data collection protocol and consent form (IRB Approval #1148461). Patients of either sex qualified for the study provided they were at least 18 years of age, had a medical history of musculoskeletal, neuropsychiatric, and/or cardiac symptoms consistent with LD, and gave written informed consent for data collection. Subjects were included in the study if they met the case definition of untreated or previously treated chronic LD with symptoms lasting more than six months, as described in detail elsewhere [23,24]. Patients were not required to have had a documented tickbite or erythema migrans rash for participation in the study because serological testing was used to detect exposure rather than active infection. De-identified patient samples were coded according to the patient’s place of residence. Blood was drawn and serum was separated at independent laboratories including BioReference^®^, LabCorp^®^, and AnyLabTestNow^®^, and serum samples were transported to the reference laboratory for immunoblot testing (see below).

A total of 175 human sera expected to be negative for Bbsl and RFB were obtained from the Centers for Disease Control and Prevention (CDC), College of American Pathologists, New York State Department of Health, New York Biologics (Southampton, NY, USA) and IGeneX Reference Laboratory (Milpitas, CA, USA). The IGeneX samples were leftover sera received for routine testing for tick-borne diseases that would otherwise be discarded. Testing of control sera was performed by laboratory personnel in a blinded fashion in the same manner as testing of clinical samples from Bbsl and RFB patients. Control sera were used to evaluate immunoblot specificity/sensitivity and were not intended as population controls for the sample cohort.

### 2.2. Antigen Preparation

Recombinant target proteins were obtained by cloning hybrid gene constructs or portions of genes into pET vectors, expressing the gene products in *Escherichia coli* (GenScript, Piscataway, NJ, USA), then isolating the proteins to >90% purity, as previously described [1,2]. Bbsl recombinant proteins were derived from several US and European species of Bbsl including Bbss strains B-31 and 297 for the detection of the following targets: P18, P23 (OspC), P28, P30, P31 (OspA), P34 (OspB) P39 (BmpA), P41, P45, P58, P66, P93, the variable surface antigen of Bbss (VlsE), and C6 (a hybrid protein containing the immunodominant region of VlsE from different Bbsl species), as previously described [1]. The targeted Bbsl species were Bbss (B-31 and 297), *B. californiensis*, *B. spielmanii*, *B. afzelii/B. garinii*, *B. mayonii*, and *B. valaisiana.* Recombinant proteins from different RFB species were derived for the detection of four target antigens, BipA, GlpQ, fHbp, and FlaB, as previously described [2]. The targeted RFB species were *B. hermsii*, *B. miyamotoi*, *B. turicatae*, and *B. turcica*.

### 2.3. Preparation of Antigen Strips

Antigen strips for Bbsl and RFB immunoblots were prepared as previously described [1,2]. In brief, purified proteins and control proteins were diluted (7–19 ng protein/line) and sprayed in straight lines on nitrocellulose sheets (Amersham Protran, GE Healthcare Life Science) using a BioDot liquid dispenser (BioDot, Irvine, CA, USA). Protein L (Sigma, St. Louis, MO, USA) and mixed human immunoglobulin M (IgM) and IgG (Sigma, St. Louis, MO, USA) were used as control proteins for detecting the addition of human serum and for detecting the addition of alkaline phosphatase conjugated anti-human antibodies. The sheets were then blocked with 5% non-fat dry milk and sliced into 3-mm-wide strips.

### 2.4. Detection of Antibody Reactivity

Serological immunoblot testing was performed at IGeneX Reference Laboratory, a high-complexity testing facility with Clinical Laboratory Improvement Amendments (CLIA) certification.

The reactivity between *Borrelia*-specific antibodies from test sera and *Borrelia* antigens on immunoblots was detected as previously described [1,2]. In brief, strips were labeled, soaked in diluent (100 mM Tris, 0.9% NaCl, 0.1% Tween-20, and 1% non-fat dry milk) for 5 min in a trough; then, a 10-μL aliquot of the test or control serum was added to the strip. Strips with sera were incubated at room temperature for one hour, washed three times with wash buffer (KPL, Gaithersburg, MD, USA) at room temperature, and the final wash solution was then aspirated. To detect IgG and IgM reactivity, strips were incubated with alkaline phosphatase-conjugated goat anti-human IgG at 1:10,000 dilution or IgM at 1:6000 dilution, respectively (KPL, Gaithersburg, MD, USA), for one hour, and then washed three times. To visualize bands of antibody/antigen reactivity, the strips were reacted with a chromogenic substrate, 5-bromo-4-chloro-3-indolylphosphatenitro-blue tetrazolium (BCIP/NBT, KPL, Gaithersburg, MD, USA), and the reaction was terminated by washing with distilled water after the calibration control produced a visible band at 39 kDa. Bands demonstrating an intensity lower than that of the calibration control were reported as negative. Human sera from patients with confirmed *Borrelia* infection were used as positive controls and sera from uninfected persons were used as negative controls. All immunoblot testing of patient samples was performed with simultaneous testing of positive and negative control serum samples.

### 2.5. Scoring of Immunoblots

For Bbsl immunoblots, the following bands (in kDa) were scored for IgG reactivity: 18, 23 (OspC), 28, 30, 31 (OspA), 34 (OspB), 39 (BmpA), 41 (FlaB), 45, 58, 66, and 93. The following bands (in kDa) were scored for IgM reactivity: 23 (OspC), 31 (Osp A), 34 (Osp B), 39 (BmpA), 41 (FlaB), and 93. Interpretation of immunoblots was determined by two different criteria: the CDC criteria, and the in-house criteria, as previously described [1]. Based on the CDC criteria, IgM reactivity was interpreted as positive if two of the three antigen bands of 23, 39, and 41 kDa were positive, and IgG reactivity was interpreted as positive if five of the 10 antigen bands of 18, 23, 28, 30, 39, 41, 45, 58, 66, and 93 were positive. Based on the in-house criteria, IgM reactivity was considered positive if two out of the four bands of 23, 31, 39, and 41 kDa were present, and IgG reactivity was considered positive if two out of the six bands of 23, 31, 34, 39, 41, and 93 kDa were present.

**Figure A1:**
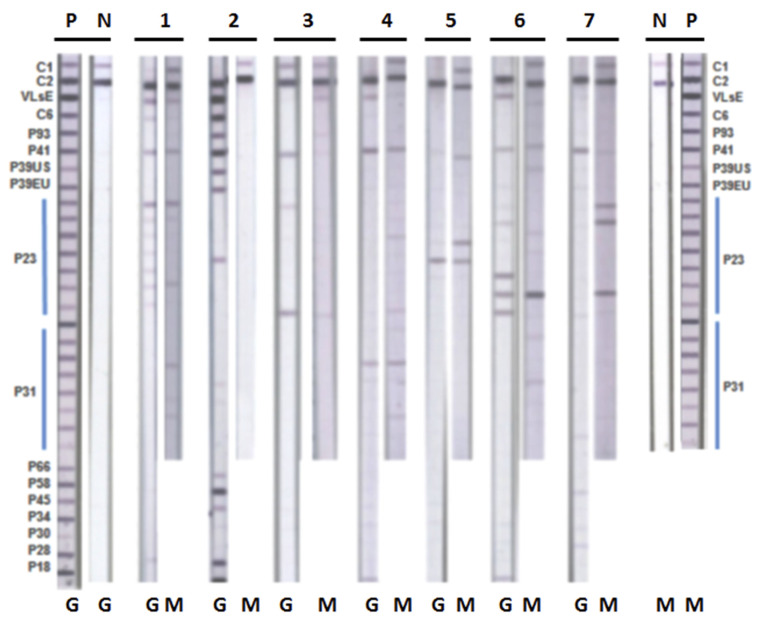
Immunoblot reactivity for *Borrelia burgdorferi* sensu lato (Bbsl). Immunoblot strips with recombinant *Borrelia* antigens were incubated with representative patient serum samples, as described in Section 2. Blots were interpreted according to IGeneX criteria (see text). P—positive control; N—negative control; G—Bbsl immunoblot immunoglobulin G (IgG); M—Bbsl immunoblot IgM; Lane 1—*B. burgdorferi* B-31 positive IgM and IgG; Lane 2—*B. mayonii* positive IgG; Lane 3—*B. speilmanii* positive IgG; Lane 4—*B. californiensis* positive IgM and IgG; Lane 5—mixed infection *B. mayonii* and *B. californiensis* positive IgM; Lane 6—*B. burgdorferi* European species positive IgM and IgG; Lane 7—Bbss and *B. burgdorferi* European species positive IgM.

**Figure A2:**
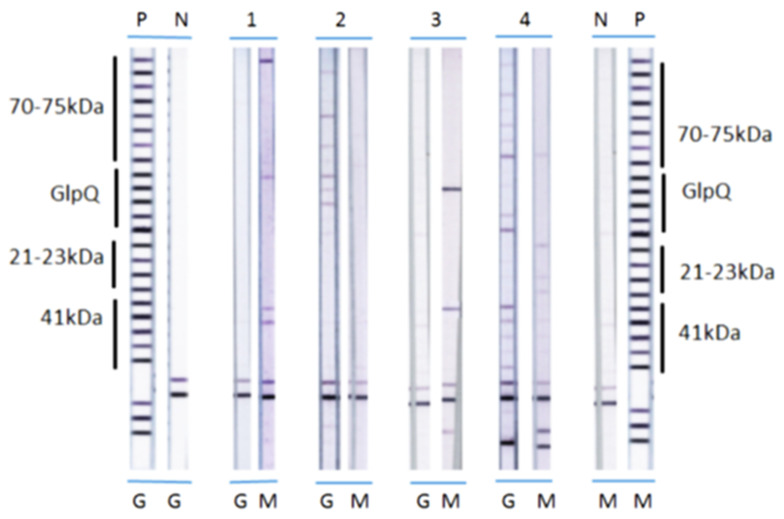
Immunoblot reactivity for Relapsing Fever *Borrelia* (RFB). Immunoblot strips with recombinant *Borrelia* antigens were incubated with representative patient serum samples, as described in Section 2. P—positive control; N—negative control; G—RFB immunoblot IgG; M—RFB immunoblot IgM; 1—*B. hermsii* positive IgM; 2—*B. turicatae* positive IgG; 3—*B. miyamotoi* positive IgM; 4—*B. turcica*-like positive IgG.

For RFB immunoblots, detection of either IgG or IgM antibodies against FlaB, as well as any two out of the three antigens BipA, GlpQ, and fHbp, gave the best specificity for RFB species. Detection of either IgG or IgM antibodies to one or more proteins within each antigen type was regarded as a positive reaction for that antigen type. Applying the same criteria for reactivity of either IgM or IgG antibodies to the four RFB antigens led to optimum sensitivity of detection, as previously described [2]. Immunoblot reactivity for Bbsl and RFB in representative patient serum samples is shown in Figure A1 and Figure A2 (Appendix A).

## 3. Results

Our cohort consisted of 90 patients who met the clinical case definition of chronic Lyme disease (CLD), as recently described [24]. Seventy-seven patients resided in the state of California and 13 resided in Mexico. Immunoblot testing revealed that all 90 subjects with suspected LD in our cohort were seropositive for *Borrelia*; a total of 42 patients (47%) were seropositive for Bbsl (Group 1), and a total of 56 patients (62%) were seropositive for RFB (Group 2). Thirty-four patients (38%) were seropositive for Bbsl alone, 48 patients (53%) were seropositive for RFB alone, and eight patients (9%) were positive for both Bbsl and RFB. The test results of these subjects in terms of place of residence, species of *Borrelia* seroreactivity, and IgM/IgG reactivity are summarized in Table A1 (Appendix A).

Group 1 consisted of patients seroreactive for Bbss (14), *B. californiensis* (eight), *B. spielmanii* (10), *B. afzelii/B. garinii* (10), and mixed infections that included *B. mayonii* (three). Group 2 included patients who were seroreactive for *B. hermsii* (nine), *B. miyamotoi* (seven), *B. turicatae* (nine), and *B. turcica* (two). Results for Group 1 and Group 2 seroreactivity are summarized in Table A2 (Appendix A). Sera from four patients in Group 1 were seropositive for two or more species of Bbsl, while sera from two patients in Group 2 were seropositive for two species of RFB. Seroreactivity to the exact *Borrelia* species in the remaining Group 1 and Group 2 patients could not be defined using the immunoblot technique.

As shown in Table A1 (Appendix A), 14/42 patients (33%) in the Bbsl group had antibodies to Bbss based on reactivity with Bbss-specific antigens derived from the B-31 and/or 297 strains. Four had antibodies to Bbss only, while the remaining 10 patients reacted with Bbss and other Bbsl species. In five samples (8, 12, 51, 86, and 88), the signal intensity with multiple species including Bbss was strong. In the remaining five samples (22, 31, 52, 63, and 64), the signal with other species was much stronger than Bbss.

The results obtained with the 175 control sera that were expected to be negative for Bbsl and RFB yielded a false positive rate of 2.3% (4/175 samples) for the Bbsl immunoblot and 2.9% (5/175 samples) for the RFB immunoblot (Table A3, Appendix A). False positive tests for Bbsl were seen with a healthy endemic serum (one control), viral infection (one control), and syphilis (two controls). False positive tests for RFB were seen with an allergy patient serum (one control), multiple sclerosis (one control), viral infection (one control), and syphilis (two controls).

## 4. Discussion

Using the new immunoblot test, our pilot study of patients residing in California and Mexico who met the CLD case definition revealed that all had exposure to either Bb or RFB species. Positive immunoblots were further characterized at the species level for the following Bbsl: Bbss, *B*. *californiensis*, *B. spielmanii*, *B. afzelii/B. garinii*, and *B. mayonii*; they were also characterized at the species level for the following RFB: *B. hermsii*, *B. miyamotoi*, *B. turicatae*, and *B. turcica.* Most sera were reactive to either Bb spp. alone (38%) or to RFB spp. alone (53%), with few seropositive to both Bb spp. and RFB spp. (9%). The lack of widespread dual reactivity suggests that cross-reactivity in our immunoblots between RFB and Bb spp. is unlikely. Immunoblot testing of control sera demonstrated excellent specificities of 97.7% for the Bbsl assay and 97.1% for the RFB assay (Table A3, Appendix A). In addition, our data indicate that, in California and Mexico, exposure to RFB spp. appears to be more common than exposure to Bb spp., and this distribution is consistent with published distribution maps [13].

Spirochetes falling into the Bbsl complex are distributed worldwide, with most cases of LD reported in the USA, Europe, and Asia [8,25]. The CDC states that approximately 30,000 cases of LD are reported in the USA each year using surveillance criteria featuring two-tier Bbss testing; however, when tracked using other methods, it is estimated that more than 300,000 people develop LD in the USA annually [26,27]. The fact that CDC surveillance criteria featuring two-tier Bbss testing captures less than one out of every ten cases shows that LD is underreported, and the results of our study suggest that some people with suspected LD who fail to meet surveillance criteria may be infected either by Bbsl or by RFB that are not cross-reactive with Bbss on two-tier testing.

The immunoblot testing used in our study enabled differentiation of Bbsl into six categories: Bbss, *B. californiensis*, *B. spielmanii*, *B. mayonii*, the European species *B. afzelii/B. garinii*, and undifferentiated Bb spp. Based on surveillance reporting in the USA, the distribution pattern of Bbss is characterized by human cases reported in all 50 states with the majority reported in the Northeast, upper Midwest, and northern California [26,27]. However, other Bbsl species are not detected by commercial testing in the USA. Until recently, Bbss, *B. garinii*, and *B. afzelii* were considered to be the only etiologic agents of LD. Currently, nine species are said to have pathogenic potential: *B. afzelii*, *B. bavariensis*, *B. bissettii*, Bbss, *B. garinii*, *B. kurtenbachii*, *B. lusitaniae*, *B. spielmanii*, and *B. valaisiana* [28]. Nine other species including *B. californiensis* were not isolated from humans [28]. *B. afzelii*, *B. garinii*, and *B. spielmanii* are considered to be *Borrelia* species primarily found in Eurasia, while *B. californiensis* is considered to be a North American species [28]. Our understanding of the pathogenicity of *Borrelia* species is evolving, and some species that were not isolated from humans and are not considered to be pathogenic may be capable of causing illness.

The immunoblot testing used in our study enabled differentiation of RFB into five categories: *B. hermsii*, *B. miyamotoi*, *B. turicatae*, *B. turcica* and undifferentiated RFB spp. RFB are found worldwide and are a significant cause of morbidity and mortality, particularly in impoverished African countries [13]. RF caused by *B. crocidurae* was recently reported to be the most common tickborne infection affecting the human population in Senegal, and, in a cohort of 206 febrile patients, 13% were determined to have detectable *B. crocidurae* DNA in blood samples [29]. In northwest Morocco, *B. hispanica* infection was found to be responsible for over 20% of unexplained fever cases [30]. Although the ecology and epidemiology of RFB in Africa is well described [13], advances in molecular testing led to widespread identification of *Borrelia* spp. in human specimens, thus challenging prevailing thought regarding geographic distribution and prevalence of RFB infection [15]. For example, a recent study reported that approximately 23% of a cohort of 301 patients in Russia with confirmed *Borrelia* infection demonstrated positive molecular testing for *B. miyamotoi* [31]. RFB is also a growing concern in the Western USA, Central America, and South America [13,32].

RF is not reportable nationally in the USA, and there is no standard case definition [33]. In 2011, RF was reportable in 12 Western states, yielding 504 cases, with 70% of the cases reported in three states: California (33%), Washington (25%), and Colorado (11%) [17,33]. In the USA, most human cases of RF are said to occur in the Western states, and the species causing disease include *B. miyamotoi, B. hermsii*, *B. lonestari*, *B. parkeri*, *B. turicatae*, and *B. mazzotii* [4,5,17,33,34,35,36,37,38]. Most cases in the USA are caused by *B. hermsii*, transmitted by *Ornithodoros hermsi* ticks that typically live in rodent nests in coniferous forests at elevations between 1500 and 8000 feet [17,33]. These soft ticks are nocturnal rapid feeders and often go unnoticed when they bite humans during sleep [16,17]. In California, *B. miyamotoi*, *B*. *hermsii*, and *B*. *parkeri* human infections were reported [32], and *B*. *coriaceae* was detected in ticks, although human infection was not confirmed [32,39]. *B. miyamotoi* was originally detected in Japan in 1995, but it is thought to have recently arrived in the Western hemisphere [38,40]; in the USA, it was detected in the North American ixodid ticks *I. scapularis* and *I. pacificus* [35,38,41,42].

The genetic diversity of *Borrelia* spirochetes, as well as the symptoms of infection that are as diverse as the organisms causing them, makes it challenging to diagnose *Borrelia*-associated disease [15,43]. It is important to recognize that classification is a human concept and the organisms encompassing the genus *Borrelia* fall within a continuous spectrum of organisms rather than into well-defined genetically distinct groups that are easily categorized. Some species that genetically fall into the RFB group, such as *B. miyamotoi* and *B. lonestari*, are vectored by ixodid ticks rather than argasid ticks, and there is, therefore, a proposal to classify them into a new group—hard tick-borne relapsing fever (HTBRF) [4,13,38,42,44]. *B. miyamotoi* demonstrates genetic differences in accord with different geographic regions and different vectors, leading to the proposal to establish grouping into a *B. miyamotoi* sensu lato complex [14,35,45,46,47,48,49,50]. 

In addition, two recently described *Borrelia* species of unknown pathogenicity, *B. turcica* and *B. tachyglossi*, are vectored by the same ixodid ticks as Bbsl, yet genetically they more closely resemble spirochetes of the RFB complex. The genomes of these two RFB species also contain genes orthologous to Bbsl-specific genes, such that these organisms demonstrate a phenotypic link between the Bbsl and RFB *Borrelia* groups [51]. To further complicate classification, moving Bbsl into its own genus, *Borreliella*, was recently proposed; however, because the Bbsl and RFB groups share infectious and biological similarities—both are bacterial zoonoses causing illness in humans and animals, are vectored by hematophagous arthropods, and are almost identical microscopically—a split of the well-established *Borrelia* genus might create confusion among medical, scientific, and lay populations and remains controversial [52,53]. 

Another controversial issue is the transmission time that is required for ticks to infect humans with *Borrelia* species. Although older animal models suggested that transmission of Bbss required 24–48 h of tick feeding, more recent reports of animal and human *Borrelia* infection challenged this dogma [54,55,56,57,58]. Furthermore, transmission of RFB may occur significantly faster than Bbsl, requiring seconds to a few hours in animal models and humans [59,60,61]. Thus, RFB may pose a greater risk of infection to human populations due to more rapid transmission from ticks.

A case definition for chronic LD was recently proposed that includes all members of the *Borrelia* spirochete complex that would encompass both the LD and the RFB groups [24]. As the genus *Borrelia* comprises both Bb and RFB in a continuum of genetically related organisms that overlap ecologically and cause an array of similar clinical manifestations, a logical approach would be to adopt the definition proposed by Stricker and Fesler categorizing RFB along with Bbsl as causative agents of LD. As we demonstrated in this study, since there is variable antigenic expression in *Borrelia* spirochetes and overlapping species distribution, clinical distinction between Bbsl and RFB may be difficult given our limited testing capability. A broader definition of LD that includes all *Borrelia* species, therefore, makes sense.

This study highlights the dire need for a diagnostic approach that acknowledges the complexity and genetic diversity of *Borrelia* spirochetes. Commercially available serological testing kits, as endorsed by the CDC, are highly specific for Bbss, and have poor sensitivity [1,20,21,22,62]. The CDC case definition for LD is narrowly drawn and the laboratory criteria needed to qualify as a positive case are rigid [63]. Commercial serological tests are based on antigens of just one Bbss strain, B-31, and this test protocol is, therefore, not capable of detecting antibody reactivity to *Borrelia* species and strains that lack cross-reactivity with B-31—a limitation that excludes detection of many *Borrelia* pathogens [1,15,43]. 

Bb and RFB immunoblots have unique band patterns and, therefore, serological tests based on Bb antigens do not adequately detect RFB antigens [1,15,43]. Testing for *Borrelia* spirochetes should ideally encompass the full spectrum of organisms, including both Bbsl and RFB. Testing for RFB is currently available in only a handful of national reference laboratories, academic laboratories, or specialty laboratories using microscopy and molecular assays, and serological testing for RFB using “in-house” tests are only available in a few laboratories [13,15,38,43]. Microscopy is nonspecific; spirochetes are only visible when there are high bacterial loads in the blood [13,15,43], and artefacts such as pseudospirochetes (filaments derived from erythrocytes) can be mistaken for spirochetes [15,64]. Ideally, a microscopic diagnosis should be confirmed by testing using other methodologies such as serological assays. Unfortunately, because commercially available test kits for RFB are not available, RFB infection is only sporadically detected, resulting in a lack of understanding of the geographical distribution of human exposure to RFB species [15]. In contrast, the immunoblot testing described in this report detected exposure to a wide variety of Bbsl and RFB species in our pilot study.

Both Bb-seropositive patients and RFB-seropositive patients tended to be more frequently IgM positive than IgG or dual IgM/IgG positive. Prolonged IgM reactivity was demonstrated in patients with late or longstanding LD [65,66,67]. Furthermore, IgM reactivity was demonstrated in human subjects with persistent Bb infection despite treatment with antibiotics [67,68]. Our cohort met the case description for CLD and had symptoms consistent with LD and other tickborne co-infections, such as musculoskeletal, neuropsychiatric, and/or cardiac manifestations. More recently, prolonged IgM seroreactivity was shown to occur in patients with late RFB infection [15]. Our study corroborates the findings of previous studies showing that prolonged IgM reactivity is associated with both Bb and RFB infections and suggests that these infections may be persistent. The fact that IgM reactivity in *Borrelia* infections is likely to persist long after the onset of symptoms should be recognized when designing testing protocols for diagnosis.

In summary, exposure to Bbsl and RFB is a cause for concern in California and Mexico. Bbsl and RFB infections may explain LD symptoms in patients who are seronegative for Bbss. Some patients may demonstrate dual exposure to both Bb and RFB species, further complicating diagnosis and treatment. Immunoblot testing for both Bbsl and RFB allows the detection of a diverse group of *Borrelia* serotypes and will promote better understanding of human exposure to pathogenic *Borrelia* in California and Mexico.

## Appendix A

**Table A1:** Test results of study subjects showing place of residence, species of *Borrelia* seroreactivity, and IgM/IgG reactivity. CDC+, positive by CDC recommended surveillance criteria; +, positive by IGeneX in-house criteria; −, negative; IND, indeterminate.

#	State/Country	Bb IgM	Bb IgG	Bb IgM and IgG	RFB IgM	RFB IgG	RFB IgM and IgG	Species	Bbss IgM or IgG
1	Mex	CDC+	+	+	−	−	−	*B. afzelii*/*B. garinii*	
2	CA	+	+	+	−	−	−	Bbss	+
3	CA	−	−	−	+	−	−	*B. hermsii*	
4	CA	−	−	−	−	+	−	*RFB* sp.	
5	CA	CDC+	−	−	−	−	−	*B. mayonii* *B. spielmanii*	
6	CA	CDC+	−	−	−	−	−	Bbss	+
7	CA	−	−	−	+	−	−	*RFB* sp.	
8	CA	CDC+	−	−	−	−	−	Bbsl, Bbss	+
9	CA	−	−	−	−	+	−	*B. miyamotoi*	
10	CA	CDC+	+	+	−	−	−	*B. californiensis*	
11	Mex	CDC+	−	−	−	−	−	*Bb* sp.	
12	CA	+	−	−	−	−	−	Bbsl, Bbss	+
13	CA	−	−	−	+	−	−	*RFB* sp.	
14	CA	+	−	−	+	−	−	*Bb* sp. *B. hermsii*	
15	CA	−	−	−	+	+	+	*B. miyamotoi* *RFB* sp.	
16	CA	CDC+	−	−	+	−	−	*B. spielmanii* *RFB* sp.	
17	CA	−	−	−	−	+	−	*B. miyamotoi*	
18	CA	−	−	−	+	−	−	*RFB* sp.	
19	CA	−	−	−	+	−	−	*RFB* sp.	
20	CA	−	−	−	−	+	−	*RFB* sp.	
21	CA	−	−	−	−	+	−	*B. turicatae*	
22	CA	CDC+	CDC+	+	−	−	−	*B. spielmanii* (IgM) *B. mayonii* (IgG), Bbss	+
23	CA	−	−	−	+	+	+	*RFB* sp.	
24	CA	−	−	−	−	+	−	*RFB* sp.	
25	Mex	−	−	−	−	+	−	*B. turicatae*	
26	CA	−	−	−	+	−	−	*RFB* Sp.	
27	CA	−	−	−	−	+	−	*B. miyamotoi*	
28	CA	−	−	−	−	+	−	*B. turicatae*	
29	CA	CDC+	+	+	−	−	−	*B. spielmanii*	
30	Mex	−	−	−	+	−	−	*RFB* sp.	
31	CA	CDC+	CDC+	+	+	−	−	*B. californiensis* *RFB* sp. Bbss	+
32	CA	CDC+	−	−	−	−	−	*B. afzelii*/*B. garinii*	
33	CA	−	−	−	+	−	−	*RFB* sp.	
34	CA	−	−	−	+	−	−	*RFB* sp.	
35	Mex	CDC+	+	+	−	−	−	*B. californiensis*	
36	CA	−	−	−	−	+	−	*RFB* sp.	
37	CA	CDC+	−	−	+	−	−	*B. afzelii*/*B. garinii* *B. turicatae*	
38	Mex	−	−	−	+	−	−	*RFB* sp.	
39	CA	−	−	−	−	+	−	*RFB* sp.	
40	CA	−	−	−	+	−	−	*B. turicatae*	
41	Mex	−	−	−	−	+	−	*B. turicatae*	
42	CA	−	−	−	+	−	−	*RFB* sp.	
43	CA	CDC+	+	+	−	−	−	*B. californiensis*, *B. mayonii*, *B. afzelii*/*B. garinii*	
44	CA	CDC+	−	−	−	−	−	*B. spielmanii*	
45	CA	CDC+	−	−	−	+	−	*B. afzelii*/*B. garinii* *RFB* sp.	
46	CA	−	−	−	+	−	−	*B. turicatae*	
47	CA	CDC+	CDC+	+	−	−	−	Bbss	+
48	CA	+	−	−	−	+	−	*B. californiensis* *RFB* sp.	
49	CA	−	−	−	+	−	−	*RFB* sp.	
50	Mex	−	−	−	+	−	−	B. hermsii B. turcica bipA +	
51	CA	CDC+	−	−	−	−	−	*Bb* sp. Bbss (Strong+ to all OspCs)	+
52	CA	CDC+	−	−	−	−	−	*B. spielmanii* Bbss	+
53	CA	−	−	−	+	−	−	*B. turcica*	
54	Mex	CDC+	−	−	−	−	−	*B. afzelii*/*B. garinii*	
55	CA	−	−	−	+	−	−	*RFB* sp.	
56	CA	+	−	−	−	−	−	*B. spielmanii*	
57	CA	−	−	−	+	−	−	*RFB* sp. *B. miyamotoi*	
58	CA	−	−	−	+	−	−	*RFB* sp.	
59	CA	−	−		−	+	−	*RFB* sp.	
60	CA	CDC+	−	−	−	−	−	*Bb* sp	
61	CA	−	−	−	+	+	+	*B. hermsii B. miyamotoi*	
62	CA	−	−	−	−	+	−	*B. miyamotoi*	
63	CA	CDC+	−	−	IND	−	−	*B. californiensis* Bbss	+
64	CA	+	+	+	−	−	−	*B. afzelii*/*B. garinii* Bbss	+
65	CA	CDC+	−	−	+	−	−	Bbss *RFB* sp.	+
66	Mex	−	−	−	−	+	−	*B. hermsii*	
67	CA	CDC+	−	−	IND	IND	−	*B. spielmanii* *B. afzelii*/*B. garinii*	
68	CA	−	−	−	+	−	−	*RFB* sp.	
69	CA	−	+	−	−	−	−	*B. californiensis*	
70	CA	−	−	−	−	+	−	*B. turicatae*	
71	CA	−	−	−	−	+	−	*B. turicatae*	
72	CA	−	+	−	−	−	−	*B. spielmanii*	
73	CA	−	−	−	−	+	−	*B. hermsii*	
74	CA	−	−	−	+	−	−	*B. hermsii*	
75	CA	−	+	−	−	−	−	*Bb* sp.	
76	Mex	−	−	−	+	−	−	*RFB* sp.	
77	CA	−	−	−	−	+	−	*B. hermsii*	
78	CA	−	+	−	−	−	−	*B. californiensis*	
79	CA	−	−	−	−	+	−	*B. hermsii*	
80	CA	−	−	−	−	+	−	*RFB* sp.	
81	Mex	−	+	−	−	−	−	*B. afzelii*/*B. garinii*	
82	Mex	−	+	−	−	−	−	*B. spielmanii*	
83	CA	−	+	−	−	−	−	*B. afzelii*/*B. garinii*	
84	CA	−	−	−	+	−	−	*RFB* sp.	
85	CA	−	−	−	+	−	−	*RFB* sp.	
86	CA	−	+	−	−	−	−	Bbsl, Bbss	+
87	CA	−	−	−	−	+	−	*RFB* sp.	
88	CA	−	+	−	−	−	−	Bbsl, Bbss	+
89	CA	−	+	−	−	−	−	*Bb* sp.	
90	CA	−	+	−	−	+	−	*Bb* sp. *RFB* sp.	
Total		IgM only 20	IgG only 11	IgM and IgG 10	IgM only 29	IgG only 25	IgM and IgG 3		Bbss IgM or IgG 14

**Table A2:** Summary of seroreactivity for subjects in Group 1 (Bbsl), Group 2 (RFB), or both.

Immunoblot	Total
Group 1 Bbsl positive	42
Group 2 RFB positive	56
Dual Group 1 and 2 positive	8
Group 1 Bbsl positive samples	34 (38%)
Bbsl alone	8
Bbss alone	4
*B. afzelii*/*garinii* alone	6
*B. californiensis* alone	6
*B. spielmanii* alone	6
*B. mayonii* + *B. speilmanii*	2
*B. spielmanii* + *B. afzelii*/*garinii*	1
*B. afzelii*/*garinii*, *B. californiensis*, *B. mayonii*	1
Group 2. RFB Positive Samples	48 (53%)
RFB alone	25
*B. hermsii* alone	7
*B. miyamotoi* alone	4
*B. turicatae* alone	8
*B. turcica* alone	2
*B. hermsii* + *B. turcica*	1
*B. hermsii* + *B. miyamotoi*	1
Dual Group 1 and 2	8 (9%)
Bb + RFB	2
Bb + *B. hermsii*	1
*B. californiensis* + RFB	2
*B. spielmanii* + RFB	1
*B. afzelii*/*garinii* + RFB	1
*B. afzelii*/*garinii* + *B. turicatae*	1
Total cases	90

**Table A3:** Reference human sera for determining the specificity of Bbsl and RFB immunoblots.

Source	Characteristic	Total No. of Sera	Bbsl Immunoblots (+)	RFB Immunoblots (+)
CDC Reference Set (*n* = 25)	Fibromyalgia	2	0	0
	Healthy endemic	7	1	0
	Healthy non-endemic	6	0	0
	Mononucleosis	2	0	0
	Multiple sclerosis	2	0	1
	Rheumatoid arthritis	2	0	0
	Severe Periodontitis	2	0	0
	Syphilis	2	0	1
CAP and NYSHD (*n* = 42) Autoimmunity and Allergy	**Autoimmune**			
	ANA (+)	3	0	0
	ANA (−)	2	0	0
	DNA (+)	1	0	0
	Rheumatoid factor (+)	9	0	0
	Rheumatoid factor (−)	8	0	0
	**Allergy (*n* = 19)**			
	IgG (+)	13	0	1
	Spec. IgE (+)	4	0	0
	Spec. IgE (−)	2	0	0
NYB (*n* = 21) Viruses and RPR (+)	Epstein–Barr virus (EBV)	4	1	0
	Human immunodeficiency virus (HIV)	4	0	0
	Cytomegalovirus (CMV)	5	0	1
	Hepatitis C virus (HCV)	0	0	0
	RPR (+)	8	2	1
IGeneX (*n* = 87)	*Bartonella henselae* infection	7	0	0
	Human granulocytic anaplasmosis	16	0	0
	*Babesia microti* infection	14	0	0
	*Babesia duncani* Infection	41	0	0
	Human monocytic ehrlichiosis	5	0	0
	Negative controls	4	0	0
**False Positives**		**175**	4	5
**Specificity**			97.7%	97.1%

ANA—anti-nuclear antibodies; CAP—College of American Pathologists; CDC—Center for Disease Control; NYB—New York Biologics, Southampton, NY; NYSH—New York State Department of Health; RF—rheumatoid factor; RPR—rapid plasma regain test for syphilis.
